# Supplementary material for: The causal relationship between the human gut microbiota and pyogenic arthritis: a Mendelian randomization study
Source: Front Cell Infect Microbiol. 2024 Nov 26;14:1452480. doi: 10.3389/fcimb.2024.1452480 (PMC11629706; doi:10.3389/fcimb.2024.1452480)
Supplement: Supplementary file 1 [file DataSheet1.zip › mendelian test/ebi-a-GCST90027534.csv_leave_one_out.pdf]

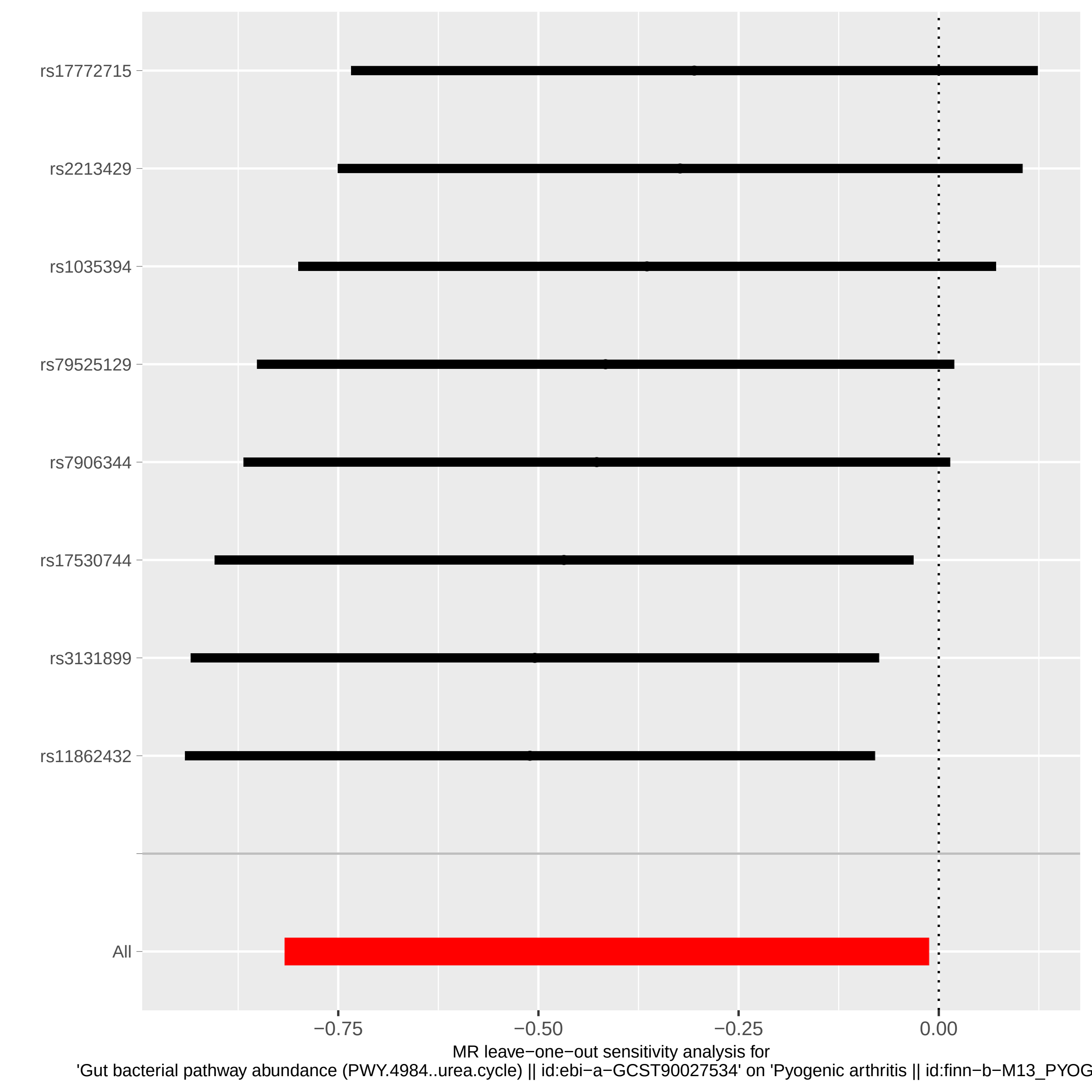
rs17772715
rs2213429
rs1035394
rs79525129
rs7906344
rs17530744
rs3131899
rs11862432
All
−0.75
−0.50
−0.25
0.00
MR leave−one−out sensitivity analysis for
'Gut bacterial pathway abundance (PWY.4984..urea.cycle) || id:ebi−a−GCST90027534' on 'Pyogenic arthritis || id:finn−b−M13_PYOG
